# Partial Degradation of Recombinant Antibody Functional Activity During Infant Gastrointestinal Digestion: Implications for Oral Antibody Supplementation

**DOI:** 10.3389/fnut.2020.00130

**Published:** 2020-08-14

**Authors:** Baidya Nath P. Sah, Jiraporn Lueangsakulthai, Bum Jin Kim, Benjamin R. Hauser, Yeonhee Woo, Amy Olyaei, Molly Aloia, Ann O'Connor, Brian Scottoline, Manoj K. Pastey, David C. Dallas

**Affiliations:** ^1^School of Biological and Population Health Sciences, College of Public Health and Human Sciences, Oregon State University, Corvallis, OR, United States; ^2^Division of Neonatology, School of Medicine, Oregon Health & Science University, Portland, OR, United States; ^3^Carlson College of Veterinary Medicine, Oregon State University, Corvallis, OR, United States

**Keywords:** palivizumab, infant digestion, human milk, antibody functional activity, respiratory syncytial virus

## Abstract

Oral administration of engineered immunoglobulins has the potential to prevent enteric pathogen-induced diarrhea in infants. To prevent infection, these antibodies need to survive functionally intact in the proteolytic environment of the gastrointestinal tract. This research examined both *ex vivo* and *in vivo* the functional survival across infant digestion of palivizumab, a model FDA-approved recombinant antibody against respiratory syncytial virus (RSV) F protein. Palivizumab-fortified feed (formula or human milk), infant gastric, and intestinal samples were incubated to simulate *in vivo* digestion (*ex vivo* digestion). Palivizumab-fortified human milk was also fed to infants, followed by collection of gastric and intestinal samples (*in vivo* digestion). Palivizumab was purified from the samples of digestate using protein G spin columns followed by filtration through molecular weight cut-off membranes (30 kDa). Palivizumab functional survival across *ex vivo* and *in vivo* digestion was determined via an anti-idiotype ELISA and an RSV plaque reduction neutralization test. Palivizumab concentration and RSV neutralization capacity both decreased when incubated in intestinal samples (*ex vivo* study). The concentration and neutralization activity of orally-supplemented palivizumab also decreased across infant digestion (*in vivo* study). These results indicate that if recombinant IgGs were selected for oral supplementation to prevent enteric infections, appropriate dosing would need to account for degradation occurring in the digestive system. Other antibody formats, structural changes, or encapsulation could enhance survival in the infant gastrointestinal tract.

## Introduction

Infectious diarrhea kills more than 2,000 children under 5 years of age every day (1–3). Breastfeeding is associated with lower infection risks in infants (4–6), and human milk enhances passive immunity of breastfed infants by supplying pathogen-specific neutralizing antibodies (7). Following the human milk model of maternal antibodies facilitating immunological protection for offspring, oral provision of pathogen-specific recombinant immunoglobulins could help prevent diarrheal infections in infants. To prevent infection, however, orally-delivered recombinant antibodies would have to resist degradation from exposure to milk and gastrointestinal proteases and pH changes (from pH 3.5–8) across the gastrointestinal tract (8, 9). Proteolytic enzymes, such as carboxypeptidases, elastase, plasmin, and kallikrein, are present in breast milk (10). These proteolytic enzymes may be active during gastrointestinal digestion, as inactive cathepsin D in breast milk is activated by the acid conditions of the stomach (11). Many digestive enzymes, including pepsin, trypsin, and chymotrypsin, also mix with feed (human milk) during infant digestion. No studies have thus far been reported for the effect of human digestive proteases on viral neutralization; however, these enzymes may degrade antibodies.

The extent to which recombinant antibodies survive across infant digestion remains unknown. Functional survival of recombinant antibodies across digestion needs to be examined to assess their potential as oral supplements to prevent enteric infections.

As a model for examining the functional survival of recombinant antibodies across digestion, we selected palivizumab (a humanized monoclonal recombinant IgG1κ), the only FDA-approved recombinant antibody for use in infants to prevent infections, and which is administered via intramuscular injection. Palivizumab recognizes and binds to the fusion protein (F) of RSV, thereby inhibiting infection of host cells (12, 13). In our previous study (14), palivizumab was not stable across *ex vivo* incubation in infant gastric and intestinal samples, whereas naturally occurring human milk RSV-specific antibodies were stable. Whether the degradation of palivizumab as measured by ELISA across *ex vivo* infant digestion corresponds with a loss of functional capacity of palivizumab to neutralize RSV remained unknown. The aim of this study was to measure the extent to which palivizumab retains functional RSV neutralization capacity across incubation within *ex vivo* infant gastric and intestinal samples (*ex vivo* digestion) and across gastric and intestinal sampling sites after oral supplementation to infants (*in vivo* digestion). This work serves as a model for examining the digestion of recombinant antibodies that can be used to inform future development of oral enteric pathogen-specific recombinant antibodies for the prevention of infectious diarrhea.

## Materials and Methods

### Digestion of Human Milk and Formula (*ex vivo* and *in vivo*)

**Figure 1:**
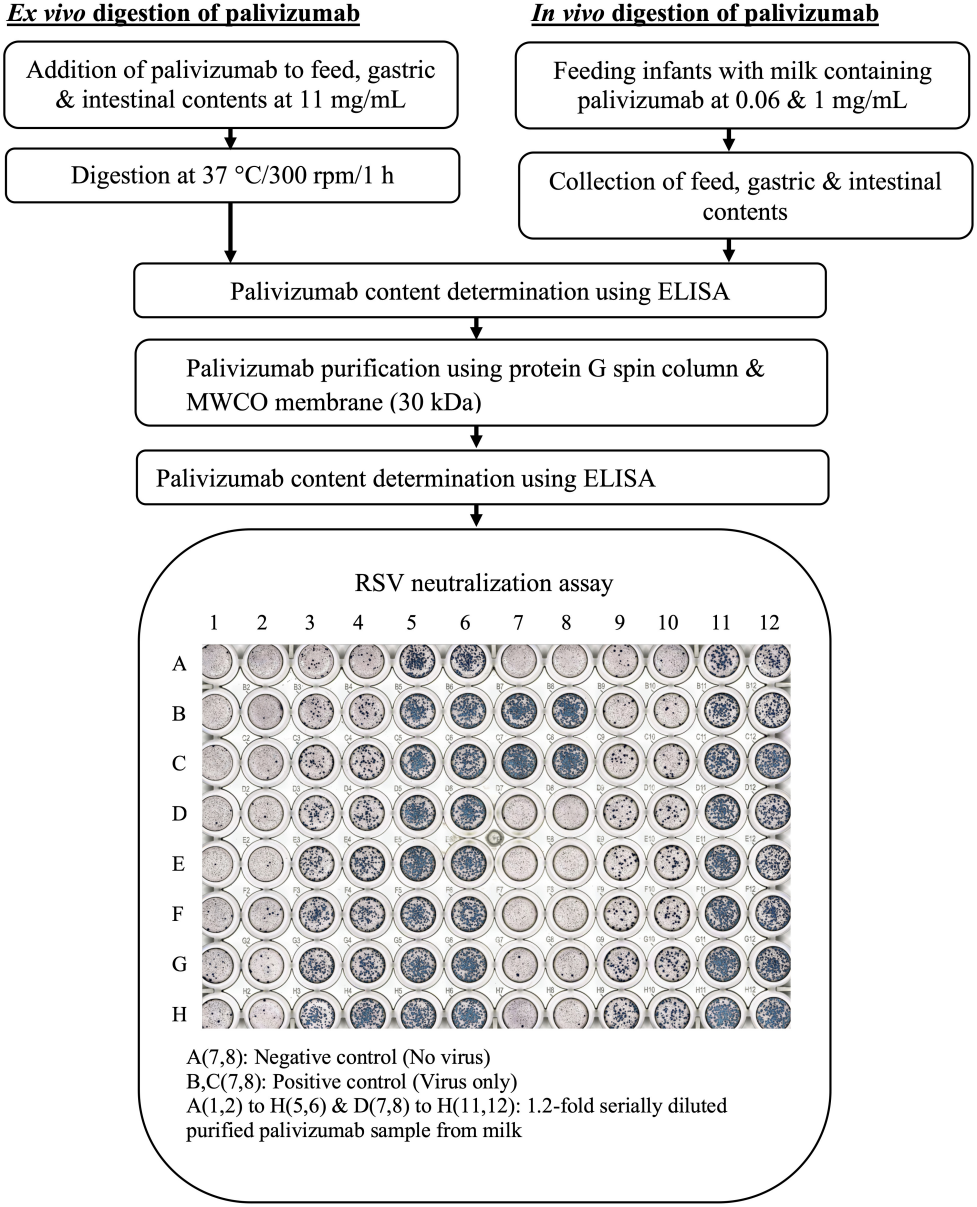
Experimental workflow for determination of degradation of palivizumab functional activity during infant digestion.

*In vivo* digestion samples were collected from infants at the Doernbecher Children's Hospital Neonatal Intensive Care Unit (NICU) located at Oregon Health & Science University in Portland, OR, after obtaining parental informed consent (Figure 1). Inclusion criteria for infants in this study were infants already admitted to the NICU, >34 weeks corrected gestational age, with an indwelling nasogastric or orogastric feeding tube and tolerating full enteral feeding volumes (typically 150–160 mL/kg/day). Exclusion criteria were infants with diagnoses that were incompatible with life, infants not being fed enterally, major gastrointestinal system anomalies affecting protein digestion, severe genitourinary anomalies, and significant metabolic or endocrine diseases. Prior to feeding, a nasally-placed tube was placed into the distal duodenum or proximal jejunum. Gastric and intestinal samples were collected from four infant pairs (Table 1). Feeds were delivered via nasogastric tubes over 30 min or less. Infants were fed without palivizumab (formula for infant 1, fortified mother's milk for infant 2) or with palivizumab (60 μg/mL in fortified mother's milk for infant 3 and 1,000 μg/mL in unfortified mother's milk for infant 4). This range of feed types represent all common feed types fed to infants in the NICU, allowing us to encompass this potential variability within the analysis of the extent of palivizumab digestion. Two milliliters of feed samples were collected in sterile vials on ice. Each infant's gastric contents (0.5–2 mL) was withdrawn by suction 30 min after completion of feeding into a 3-mL syringe and transferred in sterile vials, and placed on ice. Intestinal samples were collected from the nasojejunal/duodenal tube into sterile vials on ice via gravity flow as the digesta passed the collection tube port. Gastric and intestinal samples collected are, thus, mostly composed of the most proximal feed with the addition of digestive secretions. The sample vials were immediately stored at −80°C. The frozen sample vials were transported on dry ice to Oregon State University and stored at −80°C.

**Table 1:** Demographics of four mother–infant pairs sampled for feed (formula or human milk), gastric, and intestinal contents.

**Demographics**	**Infants^†^**
Gestational age at birth, weeks	32.08 ± 4.48 (27.1–38)
Postnatal age at feeding, days	41.50 ± 22.93 (23–75)
Corrected gestational age at feeding, weeks	38.00 ± 2.31 (36.4–41.3)
Body weight at sampling, kg	2.87 ± 0.53 (2.45–3.63)
Length at sampling, cm	46.25 ± 3.77 (42–51)
Head circumference at sampling, cm	35.50 ± 3.54 (31–39.5)
Total kilocalories intake, kcal/kg/day	131.25 ± 25.40 (108–165)
Specific feed volume, mL	43.50 ± 12.23 (30–57)

† *Values are mean ± SD (range)*.

For *ex vivo* digestion of palivizumab (Figure 1), the samples [feed (formula for infant 1 or fortified mother's milk for infant 2), gastric, and intestinal samples] were thawed quickly at 37°C with shaking at 300 rpm (~1 min). Palivizumab was added to samples (feed, gastric, and intestinal) at 11 mg/mL and digested at 37°C with shaking at 300 rpm for 1 h (feed, gastric, and intestinal samples) in an Eppendorf ThermoMixer® C (Eppendorf AG, Hamburg, Germany). The higher concentration of palivizumab used for the *ex vivo* incubation compared with the feeding study was selected to allow the use of lower sample volumes while extracting enough palivizumab for RSV plaque-neutralization assay.

### Determination of Palivizumab Content Using Enzyme-Linked Immunosorbent Assay (ELISA)

The Palivizumab content in the samples was determined using an anti-idiotype ELISA with HCA261 (Bio-Rad, Richmond, CA, USA) as a capture antibody and horseradish peroxidase-conjugated goat anti-human IgG gamma chain (STAR 106P, Bio-Rad) as a detection antibody according to the method developed by Bio-Rad, with some modifications. Briefly, 100 μL of HCA261 at 1 μg/mL in phosphate-buffered saline (PBS; pH 7.4) was added in each well of a clear flat-bottom 96-well plate (Nunc MaxiSorp; Thermo Fisher Scientific, Waltham, MA, USA) and incubated overnight at 4°C. Wells of the microplate were washed three times with 200 μL of PBS containing 0.05% Tween-20 (PBST) (Bio-Rad) and blocked for 1 h with 150 μL of PBST containing 1% bovine serum albumin (BSA) at RT. Samples were diluted 2-fold with PBST containing 1% BSA, and palivizumab standards were prepared in PBST containing 1% BSA in the range of 1–1,000 ng/mL. Palivizumab standards/diluted samples (100 μL) were added to each well after washing three times using PBST and incubated for 1 h at RT. The wells were washed three times with PBST as described above; 100 μL of horseradish peroxidase-conjugated goat anti-human IgG gamma chain detection antibody at 0.13 μg/mL PBST containing 1% BSA were added to each well and incubated at RT for 1 h. After the plates were washed 6 times with PBST as described above, 100 μL of 3,3′,5,5′-tetramethylbenzidine substrate solution (Thermo Fisher Scientific) were added to each well and incubated for 5 min. The reaction was stopped by adding 50 μL of 2 N sulfuric acid and the absorbance was measured at 450 nm using a microplate reader (Spectramax® M2, Molecular Devices, Sunnyvale, CA, USA). The samples from feed, infant gastric, and intestinal contents were tested at least two dilutions with 3 replicates of each dilution. Replicate measurements were averaged. The percentage survival of intact ELISA-detectable palivizumab at each digestion point was determined with respect to the unincubated sample in the *ex vivo* study, whereas it was determined with respect to feed in the *in vivo* study. Percentage survival was determined for each dilution (the average of three replicates) separately and these values were used for statistical analyses.

### Purification of Palivizumab Using Protein G Spin Column and 30-kDa Molecular Weight Cut Off (MWCO) Filtration

Milk, gastric and intestinal samples contain substances such as β-casein, milk fat, immunoglobulins (SIgA, IgG, and IgM), lactoferrin, proteases, protease inhibitors, lactoperoxidase, cells, and bacteria that can introduce background effects on the RSV neutralization assay (15–18). Thus, palivizumab was purified from the *ex vivo* and *in vivo* samples using protein G column and 30 kDa-MWCO filtration. Protein G spin column (Thermo Fisher Scientific) and all buffers were equilibrated to RT (30 min). Storage solution of the column was passed through by centrifuging the column at 5,000 × g, 20°C for 30 s. To equilibrate the columns, 400 μL of the Pierce™ protein G IgG binding buffer (proprietary composition, pH 5.0, containing 0.02% sodium azide) were added and the column was centrifuged at 5,000 × g, 20°C for 30 s. The equilibration step was repeated once. The volume of sample added to the protein G column varied based on the infant and sample type, and was selected based on a desired final concentration of 300 μg/mL palivizumab in the purified sample, assuming a standard 50% palivizumab loss after complete extraction (protein G and 30-kDa MWCO filtration). This allowed for a 30-fold dilution to overcome background effects in the neutralization assay while maintaining a target 10 μg/mL palivizumab starting concentration in the neutralization assay. Samples were separately diluted with the binding buffer in the ratio of 1:3 (v/v) to ensure optimal ionic strength and pH for binding. The diluted sample was centrifuged for 10 min at 1,000 × g, 4°C, and the supernatant was collected for palivizumab extraction. The pellet was dissolved in 1 mL binding buffer, centrifuged as described above, and the supernatant was collected and combined with the previous supernatant. An aliquot of this supernatant prepared from sample-buffer mixture (500 μL) was added to a protein G spin column and mixed end-over-end for 10 min and centrifuged at 5,000 × g, 20°C for 30 s. To wash the column, 500 μL of the binding buffer were added, mixed to resuspend the resin and centrifuged at 5,000 × g, 20°C for 30 s. These wash steps were repeated 9 times. To elute bound palivizumab, 500 μL of the Pierce™ gentle Ag/Ab elution buffer (proprietary composition, high ionic strength, pH 6.6) were added to the column, the column was mixed end-over-end to resuspend the resin and centrifuged at 5,000 × g, 20°C for 60 s. Elution steps were repeated 7 times. To remove remaining interfering substances, the protein G extract was added to a 30-kDa MWCO centrifugal filter unit. Prior to the addition of the sample, 5 mL of Dulbecco's Modified Eagle Medium (DMEM), without serum, were added to the device followed by centrifugation at 3,000 × g, 4°C for 3 min to wash the apparatus. This washing step was repeated once. Four milliliters of each protein G extract were combined with 5 mL of DMEM (no serum, with antibiotic), added to the MWCO device and centrifuged at 1,000 × g, 4°C for 10 min. To allow for additional removal of interfering substances, 5 mL of DMEM (without serum) were added and the MWCO device was centrifuged (repeated 2 times). The retentate (purified palivizumab) was collected, and palivizumab concentration in the purified samples was determined by ELISA. The efficiency of this extraction was not 100% and differed across sample types. To make a fair comparison, the extracted palivizumab concentrations were normalized to a specific dilution of the original concentration prior to the neutralization assay. To do so, a dilution that would bring the original palivizumab concentration close to 10 μg/mL was selected as the target for normalizing the dilution of the purified sample. Purified palivizumab samples were then diluted to reach the concentration of this selected dilution for the respective unpurified sample. This normalized dilution number was used to interpret the results of the plaque assay.

### Determination of Plaque Reduction Neutralization Titer

#### Preparation of RSV Frozen Stock

HEp-2 cells (ATCC® CCL23™) were seeded in a tissue culture flask (75 cm^2^) with DMEM containing 10% fetal bovine serum (FBS) and 1% antibacterial-antimycotic solution and allowed to grow until reaching >95% confluency (typically 24–48 h) in a 5% CO_2_ incubator at 37°C. The cell monolayer was washed three times with sterile Hank's balanced salt solution and infected with 1 mL of frozen RSV subtype A (Long strain; ATCC® VR-26™; American Type Culture Collection, Manassas, VA, USA) stock (3.74 × 10^7^ plaque-forming units/mL) in 3 mL of virus growth medium (DMEM with antibiotics-antimycotics without serum). The flask was incubated at 37°C in a CO_2_ incubator for 2 h. The flask was rocked in the North–South (N–S) and East–West (E–W) direction every 15 min to maintain an even virus distribution and avoid potentially drying the cells. After 2 h of incubation, 10 mL of the virus growth medium were added to stop virus adsorption. The flask was examined every day during post-infection incubation via an inverted microscope for cytopathic effects, namely syncytia formation, rounding and sloughing, to ensure the viral infection had taken place. After 5 days post-infection, the spent media was forcefully mixed 10 times with a pipette to free the infected, weakly attached cell monolayer from the flask and collected in a 50-mL Falcon tube. The pooled cells and supernatants were centrifuged at 280 × g, 4°C for 5 min and the supernatant was collected, leaving ~200 μL of supernatant in the tube with the pelleted cells. The cell pellet was resuspended with the leftover 200 μL of supernatant and frozen immediately on dry ice, followed by quickly thawing in a 37°C water bath. This freeze-thaw step was repeated 3 times and the tube was agitated with a vortex mixer after each cycle. All the freeze-thawed cell debris was pooled with the saved supernatant, sterile glycerol was added at 15% (v/v) and mixed well with a vortex mixer. The virus suspension was pipetted into cryovials (300 μL/cryovial) and stored at −80°C for long-term storage.

#### Determination of Neutralization Titer of Samples Against RSV

The plaque reduction neutralization assay was performed with some modifications (19). Briefly, HEp-2 cells were seeded onto a 96-well plate at a density of 3.5 × 10^5^ cells/mL in DMEM containing antibiotic-antimycotic solution (1%) and 10% FBS and grown in a CO_2_ incubator until the cells reached >95% confluency. Frozen stock of human RSV (10(6.00)TCID[50]/0.1 mL, HEp2, 2 days; 7 × 10^6^ plaque forming units/mL) was diluted 250-fold in DMEM containing antibiotic-antimycotic solution (1%) without FBS. An aliquot of the diluted virus (40 μL) was mixed with an equal volume of 1.2-fold serially diluted samples in DMEM without FBS (40 μL) in triplicate and pre-incubated for 1 h at 37°C in a CO_2_ incubator. After washing the cell monolayer three times with DMEM containing antibiotic-antimycotic solution (1%) without FBS, sample-virus mixtures (25 μL/well) were added to the plate in duplicate wells (for a total of six wells per dilution). The plate was incubated at 37°C in a CO_2_ incubator with shaking for 2 h, with intermittent manual rocking each direction (N–S and E–W) every 15 min for 1 min in a biosafety cabinet to enable non-neutralized virus to adsorb onto the cells. The virus-sample inoculum was aspirated, and 0.1 mL of overlay medium (1% methyl cellulose (Spectrum Chemical Manufacturing Corp., New Brunswick, NJ, USA) in DMEM containing antibiotic/antimycotic solution, without FBS) was added to each well and returned to the incubator. Methylcellulose fixed the virus in position to prevent RSV progeny spreading throughout the well and ensure localization of plaques. After 48 h of incubation at 37°C, the overlay was aspirated using a multichannel aspirator. The cells were fixed by adding 100 μL/well of ice-cold acetone:methanol (60:40) for 5 min and air-drying for 30 min. The non-specific sites on the cell monolayer surface were blocked by adding 100 μL/well of 3% skim milk (MilliporeSigma) for 10 min. The cell monolayer was washed three times with PBST. A drop of BLOXALL blocking solution (Vector Laboratories, Inc., Burlingame, CA, USA) was added to each well and incubated for 10 min to inhibit endogenous peroxidase, pseudoperoxidase, and alkaline phosphatase activities. The cell monolayer was washed three times with PBST. The cells were incubated with mouse anti-RSV F protein monoclonal antibodies (MilliporeSigma) at 1:1,500 in PBST (100 μL/well) for 2 h. The cell monolayer was washed three times with PBST and incubated for 1 h in a CO_2_ incubator with alkaline phosphatase-conjugated goat anti-mouse IgG antibodies (MilliporeSigma) at 1:1,500 dilution in PBST (100 μL/well). The cell monolayer was washed three times with PBST. Individual plaques were stained by adding 100 μL per well VECTOR Black alkaline phosphatase substrate (Vector Laboratories, Inc., Burlingame, CA, USA) followed by incubation at RT for 15 min to allow color development. The cell monolayer was washed with PBST. Images of the plate with plaques were recorded using a fluorescence microscope (model: BZ-X710; Keyence Corporation, Osaka, Japan), and plaques were counted manually using Fiji, an open-source image processing package based on ImageJ. Each plate had two wells without RSV (negative control). The sample dilution number for a reduction in 50% plaque neutralization compared with plaque formation in virus-only controls was referred to as 50% neutralization titer (NT_50_), and it was interpolated from the four-parameter logistic curve drawn from % plaque reduction vs. sample dilution number using GraphPad Prism software (version 8.2.1). A separate NT_50_ value was determined for each of the three experimental replicates based on the average plaque count values of the duplicate wells. The percentage functionality loss of palivizumab at a digestion point was determined with respect to the unincubated sample in the *ex vivo* study, whereas it was determined with respect to feed in the *in vivo* study. Percentage functionality was determined for each of the three experimental replicates separately and these values were used for statistical analyses.

### Statistical Analysis

All data passed the Shapiro-Wilk normality test. Unpaired *t*-tests were performed for the *ex vivo* study to evaluate significant differences between the percentage survival of palivizumab relative to time 0 based on ELISA and the RSV neutralization assay at *P* < 0.05 for each infant separately based on measurement replicates (values from at least three dilutions measured in triplicate for ELISA and three independently calculated NT_50_ values based on duplicate wells for the plaque assay). One-way analysis of variance (ANOVA) followed by Tukey Honestly Significant Difference *post-hoc* tests were conducted in the *in vivo* study to evaluate significant differences between the mean percentage survival of palivizumab relative to feed based on ELISA and the RSV neutralization assay at *P* < 0.05 for each infant separately based on measurement replicates (values from at least two dilutions measured in triplicate for ELISA and three independently calculated NT_50_ values based on duplicate wells for the plaque assay). A two-tailed Pearson's correlation test was performed to determine the correlations between percentage palivizumab stability as measured by ELISA and NT_50_ values from the RSV-neutralization assay across gastric and intestinal *ex vivo* and *in vivo* digestion. GraphPad Prism software (version 8.2.1) was used for statistical analyses.

## Results

### Survival of Palivizumab After *ex vivo* Digestion

To study palivizumab survival across simulated infant digestion, the binding activity of palivizumab was determined via an anti-idiotype ELISA and the functional neutralizing capacity via the RSV plaque-reduction neutralization test after 1 h incubation in human milk, gastric, and intestinal digestates (*ex vivo* digestion).

**Figure 2:**
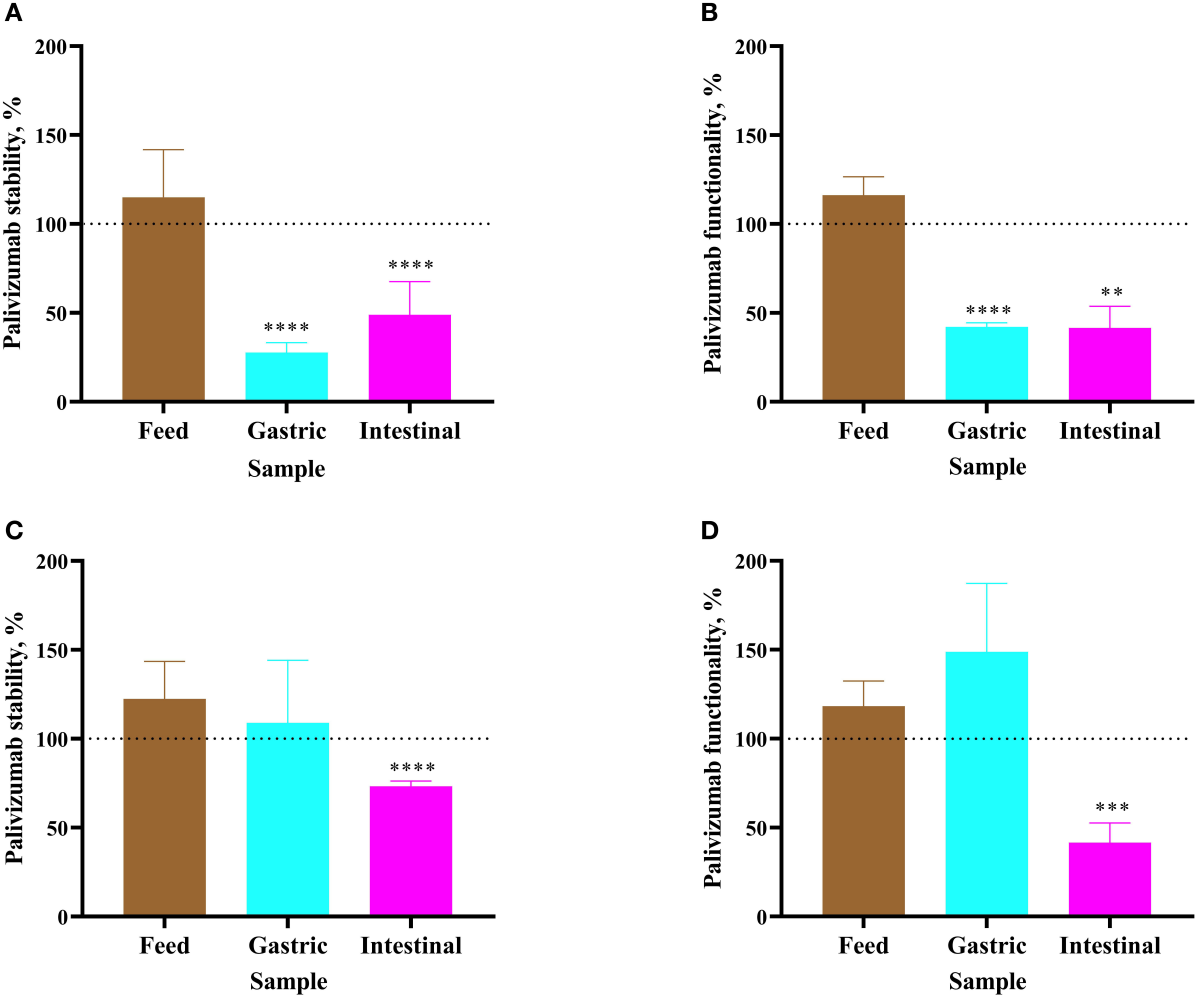
Stability of palivizumab during a 1 h *ex vivo* digestion in feed (brown bars), gastric samples (cyan) and intestinal samples (magenta) in **(A)** Infant 1 and **(C)** Infant 2, respectively, tested by anti-idiotype ELISA and represented as percentage of the original palivizumab content. Stability of palivizumab neutralization capacity across *ex vivo* digestion in the sample from **(B)** Infant 1 and **(D)** Infant 2 based on NT_50_ and represented as a percentage of the original functionality. Values are mean ± SD, *n* = 6 and 3 dilutions for Infants 1 and 2, respectively, measured in triplicate for ELISA and *n* = 3 experimental replicates measured in duplicate for the RSV neutralization assay. Asterisks show statistically significant differences (**P < 0.01; ***P < 0.001; and ****P < 0.0001) between time 0 and 1 h of incubation within each sample type using unpaired *t*-tests. The broken line shows palivizumab stability in the anti-idiotype ELISA and palivizumab functionality in the RSV neutralization assay in feed (0 h), gastric (0 h), and intestinal (0 h) as 100%.

For Infant 1, palivizumab concentration in formula remained stable after 1 h of incubation as determined by ELISA (Figure 2A). Likewise, NT_50_ remained stable (Figure 2B). Following 1-h incubation of the infant's gastric sample, palivizumab concentration decreased 72.34% (Figure 2A) and NT_50_ decreased 57.87% (Figure 2B). After 1 h of incubation of the infant's intestinal sample, palivizumab concentration decreased 51.09% (Figure 2A) and NT_50_ decreased 58.47% (Figure 2B). The combined data demonstrate that the anti-idiotype binding capacity and neutralization capacity of palivizumab was degraded during *ex vivo* gastric and intestinal digestion in Infant 1 samples. For Infant 2, both palivizumab concentration and NT_50_ were stable after 1-h incubation in fortified mother's milk and the gastric sample (Figures 2C,D, respectively). After 1 h of incubation of the intestinal sample, palivizumab concentration decreased 26.74% (Figure 2C) and NT_50_ decreased 58.43% (Figure 2D).

The combined ELISA and neutralization assay results demonstrated that palivizumab was not digested after *ex vivo* incubation in either the formula or fortified mother's milk, was variably digested in the gastric samples from Infant 1 and Infant 2 and was digested in the intestinal samples from both infants.

### Survival of Palivizumab Across *in vivo* Digestion

**Figure 3:**
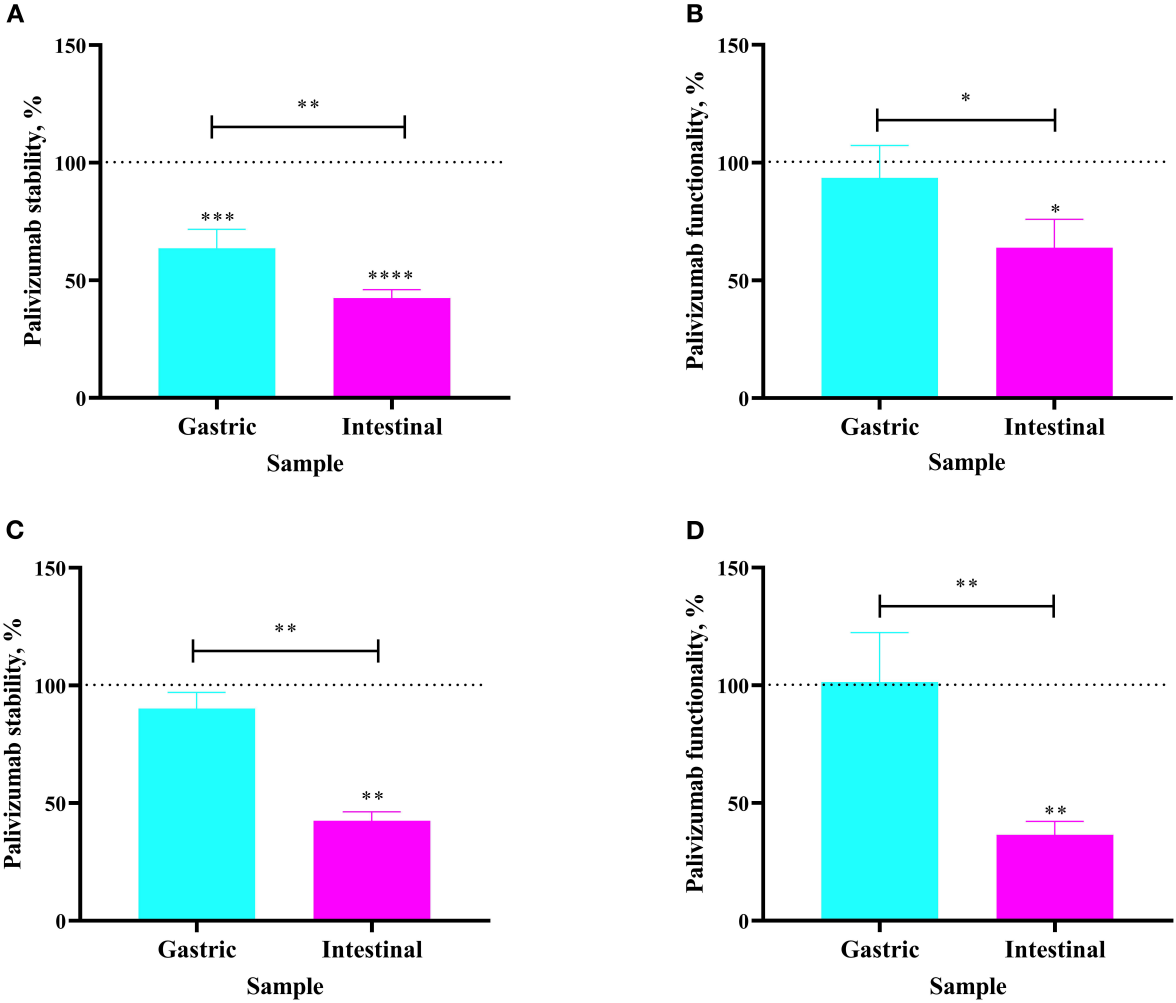
Stability of palivizumab during *in vivo* digestion in **(A)** Infant 3 and **(C)** Infant 4 tested by anti-idiotype ELISA and represented as percentage of the original palivizumab content in feed compared with the gastric (cyan bars) and intestinal (magenta) samples. Stability of palivizumab functionality during digestion in **(B)** Infant 3 and **(D)** Infant 4 tested by plaque neutralization assay and represented as percentage of the palivizumab functionality in the feed sample. Values are mean ± SD, *n* = 3 and 2 dilutions for Infants 3 and 4, respectively, measured in triplicate for ELISA, and *n* = 3 experimental replicates measured in duplicate for the RSV neutralization assay. Asterisks show statistically significant differences (*P < 0.05; **P < 0.01; ***P < 0.001, and ****P < 0.0001) using one-way ANOVA followed by Tukey's multiple comparison tests. The broken line shows palivizumab stability in the anti-idiotype ELISA and palivizumab functionality in the RSV neutralization assay in feed as 100%.

The extent to which orally-supplemented palivizumab's anti-idiotype binding capacity and RSV neutralization capacity decreased across infant digestion was examined (*in vivo* study). For Infant 3, fed 60 μg/mL of palivizumab in fortified mother's milk, palivizumab concentration was 36.39% lower in the gastric sample than in the feed (Figure 3A). The neutralization titer of palivizumab was stable during gastric digestion in Infant 3 (Figure 3B). In the intestinal sample from Infant 3, palivizumab concentration was 57.52% lower than in the feed and 21.13% lower than in the gastric sample (Figure 3A). Likewise, the NT_50_ in the intestinal sample was 36.13% lower than in the feed and 29.64% lower than in the gastric sample (Figure 3B). For Infant 4, fed 1,000 μg/mL palivizumab in unfortified mother's milk, palivizumab concentration and NT_50_ were stable in the gastric sample (Figures 3C,D, respectively). Palivizumab concentration in the intestinal sample was 57.49% lower than in the feed and 47.68% lower than in the gastric sample (Figure 3C). NT_50_ in the intestinal sample was 63.55% lower than in the feed and 65.00% lower than in the gastric sample (Figure 3D). Overall, the neutralization assay results demonstrated that palivizumab was not digested during gastric digestion, whereas it was digested during intestinal digestion of both Infant 3 and Infant 4.

We hypothesized that the functionality of palivizumab could be indicated by ELISA. A two-tailed Pearson's correlation test was performed to determine the correlations between palivizumab percentage stability as measured by ELISA and NT_50_ values from the RSV-neutralization assay across gastric and intestinal *ex vivo* and *in vivo* digestion. These variables were highly correlated (*P* < 0.0001, *r* = 0.87).

## Discussion

Diarrhea causes more than half a million deaths each year among children under 5 years old, with most deaths occurring in resource-limited countries (20, 21). Infants are born with naive immune systems, including low levels of intestinal immunoglobulin secretion (22). Feeding infants human milk significantly decreases infectious diarrhea risk, likely in part because milk provides enteric pathogen-specific antibodies (5). Infants can be protected against enteric pathogen-induced diarrhea through fortification of milk or formula with enteric pathogen-specific antibodies. To be effective in preventing enteric pathogen infection, however, oral immunoglobulins need to survive intact after exposure to the digestive system's highly degradative environment, which varies from pH 3 to 8 and contains proteolytic enzymes (8, 23, 24). The extent to which recombinant immunoglobulins remain structurally intact and functional across infant digestion remains unknown. In our previous study (14), we demonstrated that palivizumab was degraded in *ex vivo* infant gastric and intestinal digestion as observed via an RSV F protein-specific ELISA. This result contrasted with the observation that naturally occurring human milk RSV-specific antibodies remained stable across *ex vivo* digestion. As that study did not test the extent to which observed degradation corresponds with loss of RSV neutralizing capacity (i.e., functionality), herein, we examined the survival of palivizumab across *ex vivo* and *in vivo* infant digestion via a plaque reduction neutralization test in addition to an anti-idiotype ELISA.

The anti-idiotype ELISA was selected as a means to determine the extent to which palivizumab remained intact through digestion. To be detected by ELISA, both the Fab and Fc regions of the antibody would have to be sufficiently structurally intact to bind to the anti-idiotype antibody and anti-IgG antibody, respectively. To confirm the extent to which the ELISA could serve as an indicator of palivizumab functionality, we tested the neutralization capacity of palivizumab via the plaque-reduction neutralization test. To be functional in this test, palivizumab must be structurally intact enough to bind to the F protein of RSV to prevent fusion with the host cell, thereby preventing infection.

The digestion of palivizumab was tested with *ex vivo* and *in vivo* approaches. By incubating palivizumab in clinically-collected feedings (formula, fortified mother's milk, and unfortified mother's milk), and gastric and intestinal contents from neonatal intensive care unit patients, we provided conditions highly similar to those of *in vivo* digestion, including the correct concentration of enzymes. This approach more optimally mimics *in vivo* digestion than the typical *in vitro* digestion system (25, 26). Although *ex vivo* digestion overcomes some limitations of *in vitro* methods, it is a static simulation and cannot entirely replicate the dynamic complexity of human digestion. In this study, we therefore also examined *in vivo* digestion of palivizumab in infants.

Palivizumab was degraded across both gastrointestinal digestion *ex vivo* and *in vivo* as determined by ELISA and the plaque-reduction neutralization test. This loss of binding and neutralization capacity indicates that the *ex vivo* and *in vivo* gastrointestinal environments altered palivizumab structure and/or resulted in proteolytic degradation. This observed antibody degradation could result from proteolytic degradation by digestive enzymes encountered during gastrointestinal digestion and/or structural destabilization by the shift from a low gastric pH to a high intestinal pH.

The percentage stability of palivizumab based on the concentrations from the anti-idiotype ELISA and the NT_50_ values from the plaque assay were highly correlated. This correlation indicated that in future experiments, the ELISA method alone can be used as a marker of the functional activity of an antibody across digestion. This finding is an essential discovery on a level of practicality for further implementations of this research in that the ELISA method has a much higher throughput than does the RSV-neutralization assay.

A limitation of this study is the small number of infants sampled for *ex vivo* and *in vivo* digestive analysis. Though this limitation precludes analysis of the biological variation among infants, the four subjects sampled allows a clear answer to our primary research question: to what extent does a recombinant antibody survive functionally intact in the infant digestive tract. The results from both the *ex vivo* and *in vivo* analysis clearly demonstrate that the infant digestive tract degrades the functional capacity of palivizumab. Likewise, the limited numbers do not allow analysis of the effect of feed type on palivizumab digestion. However, as each infant tested herein was fed a different type of feed, we have encompassed the range of potential variability from this factor within our overall result, that palivizumab is partially functionally degraded across infant digestion.

The partial degradation of functional activities of the recombinant monoclonal antibody palivizumab against RSV suggests that use of recombinant IgG for oral supplementation to prevent enteric pathogens will require either a high degree of antibody dosing to compensate for losses during digestion, antibody encapsulation strategies, or antibody structural changes to enhance antibody stability. Future work should examine the extent to which such approaches can improve the functional survival of recombinant antibodies across infant digestion.

Pathogen-specific recombinant antibodies could have a wide array of applications within the food industry. As an example, enterally-dosed antibodies could be used either to protect against foodborne illness or to modulate the intestinal microbiome. The analytical strategies established herein would be desirable to examine the potential survival and hence, functional capacity of any such antibodies administered with foods.

## Data Availability Statement

The raw data supporting the conclusions of this article will be made available by the authors, without undue reservation.

## Ethics Statement

The studies involving human participants were reviewed and approved by Institutional Review Board of Oregon Health & Sciences University (OHSU IRB #18274). Written informed consent to participate in this study was provided by the participants' legal guardian/next of kin.

## Author Contributions

BSa performed ELISA and plaque reduction neutralization assay. BSc led sample feeding and collection. BSa, JL, BK, BH, YW, AO, MA, AO'C, BSc, MP, and DD designed the study and drafted the manuscript. BSa, BSc, and DD had primary responsibility for the final content. All authors contributed to the article and approved the submitted version.

## Conflict of Interest

The authors declare that the research was conducted in the absence of any commercial or financial relationships that could be construed as a potential conflict of interest.

## Abbreviations

RSV: respiratory syncytial virus
NT: neutralization titer
DMEM: Dulbecco's Modified Eagle Medium
ELISA: enzyme-linked immunosorbent assay
FBS: fetal bovine serum
PBS: phosphate-buffered saline (pH 7. 4)
PBST: PBS containing 0.05% Tween-20
BSA: bovine serum albumin
MWCO: molecular weight cut off.
